# Healthcare Workers' Challenges in the Implementation of Tuberculosis Infection Prevention and Control Measures in Mozambique

**DOI:** 10.1371/journal.pone.0114364

**Published:** 2014-12-15

**Authors:** Miranda Brouwer, Eliana Coelho, Carla das Dores Mosse, Luciana Brondi, Laura Winterton, Frank van Leth

**Affiliations:** 1 Health Alliance International, Technical Assistance Unit, Maputo, Mozambique; 2 Ministry of Health, Maputo, Mozambique; 3 Ministry of Health, Provincial Directorate, Tete, Mozambique; 4 Independent Consultant, Edinburgh, United Kingdom; 5 Social Anthropology, University of Edinburgh, Edinburgh, United Kingdom; 6 Department of Global Health, Academic Medical Center, University of Amsterdam, Amsterdam, The Netherlands; The George Washington University Medical Center, United States of America

## Abstract

**Objective:**

Healthcare Workers (HCWs) have a higher frequency of TB exposure than the general population and have therefore an occupational TB risk that infection prevention and control (IPC) measures aim to reduce. HCWs are crucial in the implementation of these measures. The objective of the study was to investigate Mozambican HCWs' perceptions of their occupational TB risk and the measures they report using to reduce this risk. In addition, we explored the challenges HCWs encounter while using these TBIPC measures.

**Methods:**

Focus group discussion. Analysis according content method.

**Participants:**

Four categories of HCWs: auxiliary workers, medical (doctors and clinical officers), nurses and TB program staff.

**Results:**

HCWs are aware of their occupational TB risk and use various measures to reduce their risk of infection. HCWs find it challenging to employ measures that minimize such risks and a lack of clear guidelines contributes to these challenges. HCWs' and patient behavior further complicate the use of TBIPC measures.

**Conclusion:**

HCWs in Mozambique perceive a high occupational risk of TB infection. They report several challenges using measures to reduce this risk such as shortage of material, lack of clear guidelines, insufficient motivation and inadequate training. Robust training with motivational approaches, alongside supervision and support for HCWs could improve implementation of TBIPC measures. Healthcare management should address the areas for improvement that are beyond the individual HCW's control.

## Introduction

Tuberculosis (TB) remains a serious health problem in many countries [1]. Healthcare Workers (HCWs) have higher exposure to TB than the general population and therefore have an occupational risk for TB infection [2]. The burden of TB coupled with the scarcity of trained HCWs places an additional burden on the remaining healthcare work force. TB infection prevention and control (TBIPC) aims to reduce the TB transmission risk in healthcare facilities and to lower the risk of TB infection for HCWs, patients and other facility users.

In 2009 the World Health Organization (WHO) updated its TBIPC in healthcare facilities policy [3]. The policy includes three sets of measures to prevent TB transmission grouped by level of importance. Administrative measures reducing delays in diagnosis and treatment of (presumptive) TB patients are critical first level measures in addition to educating facility users on cough hygiene. Environmental measures are the second level and include ensuring adequate ventilation aiming to reduce the amount of TB bacilli in the air. Personal Respiratory Protection (PRP) involves the use of particulate respirators by HCWs. The need for environmental measures and PRP depends on the risk of transmission in the facility. Overall managerial activities facilitate the implementation of TBIPC measures.

**Figure 1:**
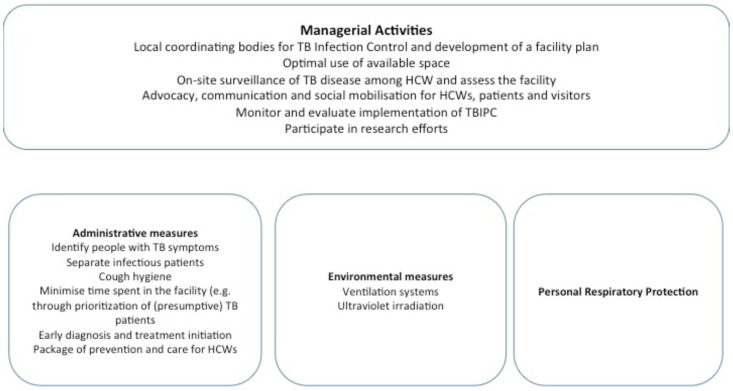
Framework for TB Infection and Prevention Control measures in Mozambique.

Mozambique is among the 22 high TB burden countries and the country's HCWs have a substantial risk for TB. The country adapted and adopted their TBIPC guidelines to the WHO's 2009 policy [4]. Fig. 1 shows the framework for TBIPC measures used in Mozambique.

HCWs are crucial in the implementation of all aspects of TBIPC measures, but are not always able to adhere to such measures. Because of the crucial role that HCW play in TBIPC we wanted to get a better understanding of how HCWs perceive their occupational risk, what measures they employ to reduce their risk to infection and the challenges they face when using these measures.

In this qualitative study we addressed the following research questions: 1) How do HCWs perceive the occupational TB infection risk? 2) What TBIPC measures do HCWs report using to prevent TB transmission? 3) What challenges do HCWs report when using such measures?

## Methods

### Setting and participants' selection

We conducted focus group discussions (FGD) in three Mozambican provinces (Manica, Sofala and Tete). In the year of the study (2010), the three provinces had TB notification rates of 146 – 363 per 100,000 inhabitants (NTP data). For the whole country it was 209 per 100,000 inhabitants. The HIV prevalence in these provinces was 7–15.5% of the 15 to 64 year old population in the three provinces and 11.5% in the country [5].

Eighty-six participants, with a minimum of five participants per FGD, participated in eleven FGD. Participants were from four categories of HCWs: auxiliary workers, medical (doctors and clinical officers), nurses and TB program staff. We choose these categories of HCWs because they represent the full range of healthcare cadre with different levels of pre- and in-service training. The categories of HCWs have different responsibilities and may have different perceptions and practices related to TBIPC. In addition, we anticipated that HCWs would participate freely in the discussions when among peers.

Provincial TB coordinators, who were part of research team, approached all healthcare facilities in area apart from the provincial hospitals to invite staff of the various categories to participate in the FGD. HCWs were at liberty to decline the invitation and all facilities were represented in the FGD. The provincial hospitals were excluded because they function very differently from the urban facilities and therefore staff may have different perceptions and practices.

### Process of focus group discussions

All eleven FGD were conducted in Portuguese in a period of two weeks in September 2010. Each FGD began with introductions of participants and researchers followed by the informed consent procedure. Thereafter digital recording started and the discussions ended when all topics were discussed. The research team briefly discussed each discussion afterwards to prepare for the next FGD. There was no need to change the FGD guide. After the eleventh FGD the team agreed that no new viewpoints had emerged and that saturation was reached.

The discussions were held outside the participants' workplace. Two experienced moderators led each discussion using a standard guide (available as S1 File). One investigator observed the discussion. Participants were given the opportunity to ask questions throughout the process.

Participants discussed what they considered to be the most important TBIPC measures while the observer listed them to organize them into relative importance of the measures according to the group's discussion.

### Data analysis

The discussions were transcribed verbatim. We performed the analysis according to the content method, which included the following steps after transcription: identification of the relevant parts of the transcripts, elaboration of analysis codes, coding of text and analysis between codes. After identification of the relevant parts of the transcripts, we developed a set of initial codes using the framework presented in Fig. 1. Subsequent coding of all transcriptions identified additional codes. The team then discussed all codes again and agreed on the final code set for the final coding and analysis. The next step in the analysis consisted of grouping of codes into overarching themes representing the three research questions. We established relationships between themes through an iterative process of examining and re-examining the relevant parts. We used Microsoft Excel for Mac version 14.3.8 in order to facilitate sorting and searching the data. Quotes representing the themes that emerged were selected for inclusion in the manuscript.

### Ethics Statement

The protocol study was approved by the Mozambican Ministry of Health National Bio-ethic Committee, while provincial health authorities provided administrative approval. After an explanation of the study all participants signed an informed consent form. The University of Washington approved the study for Health Alliance International.

## Results

Table 1 shows characteristics of the participants. The main themes that surfaced during the FGDs were the participants' perception of TB transmission risk at work, the various TBIPC measures and the challenges participants reported while using these measures. In addition there was discussion on the need for training on TBIPC to improve implementation and use of TBIPC measures.

**Table 1:** Characteristics of participants of the focus group discussions.

Healthcare Worker category	Male	Female	Median age in years (range)	Median years in service (range)	Received TBIPC training (%)	Formal classroom training	On-the-job training
Auxiliary	5	22	41(23–52)	15 (1–28)	6 (22)	4	2
Medical*	13	8	33 (22–56)	5 (0.3–30)	13 (62)	11	1
Nurses	8	10	46 (27–51)	26 (5–34)	6 (33)	3	3
TB staff	9	11	45 (28–57)	22.5 (5–35)	13 (65)	13	0
All participants	35	51	41(22–57)	15 (0.3–35)	38 (44)	31	6

*Missing data on formal training for 1 medical staff.

^§^ TBIPC = Tuberculosis Infection Prevention and Control.

### Participants' perception of the risk of TB transmission

The majority of the participants were aware of their occupational risk for TB transmission, and this risk was noted to exist across all departments in healthcare facilities. Participants perceived that those employed in TB clinics and wards, laboratories and outpatients departments (OPD) were at increased risk. Nurses working at the OPD mentioned they might be at higher risk than TB clinic staff because they were less aware of the risk and, therefore, less prepared to protect themselves. *“We, who do not routinely see TB patients, we are much more susceptible to contract TB compared to those who work with TB patients because those are already diagnosed and on treatment.” (Nurse)*

Participants expressed concern over acquiring TB at work and inadvertently infecting their families and other users of the healthcare facility. *“We are contracting the disease and I am going home without knowing I contracted the disease; I fall ill or infect my child.”(Auxiliary worker)* Several participants reported knowing colleagues that had had TB. “*And there are colleagues who already contracted TB.” (Medical)*

All participants reported they had no methods of protecting their families and themselves in the community. Most participants recognized that the frequency with which they encountered TB at work, made the work place riskier than home. A group of medical staff, however, argued that the risk at work could be easily managed because they were aware of it and had the opportunity to protect themselves.

All groups reported that the use of TBIPC measures outside HF was also important. The use of TBIPC in the communities would reduce the risk in healthcare facilities. *“If the community is informed. […] when the patient arrives in the healthcare facility, he does not cough the way he wants, he needs to have cough hygiene.” (Medical)*

### TBIPC measures that participants reported using

The TBIPC measures that participants used were cough hygiene, health education, early diagnosis and completing treatment, prioritization of patients with cough, separation, ventilation and the use of respirators. One group (nurses) mentioned the positioning of HCWs and patients in the room. These measures covered all measures in the TBIPC framework (Fig. 1) apart from early treatment initiation, a package of prevention and care for HCWs and ultraviolet irradiation though participants mentioned that sunlight should be able to enter the room.

Participants discussed what they considered the most important of these measures. All categories of HCWs reported health education among the most important measures. All categories but the nurses reported cough hygiene and all categories but the TB staff reported use of respirators by HCW among the most important measures. Measures reported by only one category were prioritization of patients with cough (auxiliary staff) and ventilation (medical staff).

### Challenges using TBIPC measures

The participants discussed their personal and their patients' ability to use the TBIPC measures. Participants encountered two significant causes for their own non-use. The first was the healthcare system, including infrastructure and the availability of necessary materials or equipment. The consultation and waiting rooms in some facilities were of inadequate space and not all windows could open. *“Our physical space is small. […] There are no fans, nothing.” (Medical)* Participants noted a conflict existed regarding the measure to use ventilation by opening doors and windows and maintaining patient privacy by closing doors during consultations. *“To respect confidentiality, you have to close the door when you talk to patients because other patients are nearby.” (TB staff)*

There was insufficient guidance as to how to apply TBIPC measures. An example given included the TBIPC measure to separate TB patients. *“In the past, all patients identified with TB here had to occupy a separate room, one did not mix them with others; then, more recently, this practice was considered discriminatory, he or she is a patient like any other patient and then we started to mix them with others. Therefore one doesn't know what is the correct criteria to be used here.” (TB staff)*

Participants reported a lack of necessary material or equipment such as respirators, boots and other protective equipment. When there was a shortage of respirators, the TB program received them first. *“There is priority to give respirators to the TB program, isn't it? We, the others, use those of paper that do not protect at all*.” (*Medical*)

Participants mentioned that the irregular supply led to indifference and developing poor practice in respirator use. *“It should be there all the time. Because if the material is there today, but finished tomorrow, I am not interested, I am not protected at all […] do I not contract the bacteria when I don't have the material?” (TB staff)*

Another reason for participants' non-use of TBIPC was related to change of existing work practices. Participants mentioned it was difficult to apply the required new practice such as using respirators consistently. *“If we had the habit to use respirators, it would be great, however, this habit does not exist.”* (*Nurse*)

Participants found that the respirators were uncomfortable to use. “*When it is very warm, a person feels a person feels suffocated”. (Medical)* In addition, the respirators also acted as a barrier or distancing mechanism when interacting with patients. They disrupted communication between HCWs and their patients. Patients felt stigmatized by HCWs who wore masks, therefore creating the illusion that the HCW lacked compassion. *“He can use the respirator, but at the same time he can't because the patient start saying that that person despises me.” (Auxiliary worker)*

Participants were aware that they needed the collaboration of patients in order to effectively implement TBIPC. They mentioned two major challenges related to patients' collaboration. The first challenge was related to patients' every day's routines and socioeconomic factors. Patients' every day's routines made it difficult to adhere to certain measures; for example, families accustomed to eating meals together found it hard to separate during these daily routines. A patients' socioeconomic situation made adherence to daily treatment at the facility difficult. Mozambique's law stipulates that a TB patient is allowed two months leave from work, however, many private companies did no comply with this regulation. As a result patients might not even take the time off to come for their medication. *“She said, “My job is in the private sector, if I have to remain at home two month, I'll stay without a job, so you nurse will give me food? […] I have to work because I can not kill my children” that is how the patients talk.” (TB staff)*

The second reason was patient behavior. Patients practiced cough hygiene by covering their mouths with their hand or a piece of cloth. Some brought a small tin with sand to cough in, adhering to health education messages of the HCWs. *“Our education also has effect. […] Because if they do not comply with our information, I think we would have spit all around.” (Nurse)*. Some patient used certain behaviors to be prioritized at the clinic and jump the queue. *“All of a sudden the majority is coughing.”* (*Medical*)

Participants used various coping strategies to deal with the challenges when using TBIPC measures. Auxiliary staff used their own boots if they did not receive those from the facility. Patients received medication outside the consultation rooms in the open air with better ventilation. “*The patient takes the medicine outside, usually we put a table outside.” (Auxiliary staff)* If participants had to wear a respirator for long, they would take a break outside the room or open the respirator slightly. “*One can retire a bit to the open air, to refresh your lungs with some oxygen.” (TB staff)*

### Training on TBIPC

Formal TB training was targeted at TB staff; however, participants felt that other staff could also benefit from such training because presumptive TB patients usually are initially seen at OPD by a general HCW.

Some facilities organized clinical sessions, which seemed to depend on the facility's management. The function of these sessions was broad: from introducing new guidelines to discussing patients. Participants did not agree on whether all categories of staff could attend the sessions. The auxiliary staff stated they did not participate in clinical sessions whereas other participant categories said they could and should. The auxiliary staff expressed concern about their lack of training. They felt devalued compared to clinical staff when it came to access to training. *“A lot of training is given to nurses, clinical officers, the doctors but the auxiliary staff does not have.” (Auxiliary staff)*

Auxiliary staff also felt placed at the bottom of the hierarchical HCW ladder. They could not approach non-auxiliary colleagues who were not observing TBIPC measures. They felt disempowered to address their concerns with the management because they thought management would not listen to them. *“Who is going to hear us, who am I?” (Auxiliary staff)*

## Discussion

The results of our study show that participants perceive TB infection as an occupational risk. They use various TBIPC measures, of which they thought cough hygiene, health education, prioritization of patients with cough, ventilation, and the use of respirators most important. The challenges in using the measures are healthcare system related such as lack of clear guidelines and insufficient material and equipment. Participants and patients' behavior also affect adequate use of TBIPC measures.

The perceived occupational risk is similar to what Ethiopian HCWs and South African nurses perceived [6] [7].

The participants were quite comprehensive in the TBIPC measures they reported using compared to the framework (Fig. 1). Though participants did not mention early treatment initiation, the importance of completing treatment and the challenges that patients may face to complete treatment, were discussed. A package of prevention and care for HCWs is not a measure that HCWs implement themselves. Ultraviolet irradiation is not used at all in the healthcare facilities where participants came from. However, they did mention the importance of sunlight. Opening windows was not only relevant for ventilation purposes but also to allow sunlight to enter. Though direct sunlight may have a role in surface decontamination, the role of sunlight in TBIPC is far from clear [8]. There was only one healthcare manager among the participants hence the absence of managerial activities in the discussions.

There was substantial agreement between the different categories of HCWs. Given the different roles and responsibilities of the HCWs, we had anticipated more variety in what HCWs reported on the use and challenges of TBIPC. This may have been because most participants came from relatively small healthcare facilities where HCWs of all levels work more closely together.

Though guidelines were available, our results show that these were not always easy to implement in daily work practice. Other studies reported similar challenges. In four West African countries, important administrative measures such as prompt identification of (presumptive) TB patients happened in less than 50% of the facilities assessed [9]. Regarding environmental measures, an Ugandan study found less than 50% of the waiting rooms adequately ventilated [10]. HCWs in a Russian study did not like using respirators because they were of poor quality [11]. Similarly, these studies found, that HCWs and patients resisted certain recommended changes in behavior, which also formed a barrier to adequate use of TBIPC measures.

TBIPC measures in healthcare facilities aim to reduce TB transmission. Mozambique and other high prevalent TB, resource-limited settings do not monitor TB disease or infection among HCWs routinely. HCWs may be more willing to use TBIPC measures when they recognize the benefits of the measures. Furthermore, implementation of TBIPC may improve when the measures are easily applicable in daily work practice, or if that is not possible, practical alternative measures are made available. Our study showed that it frustrated HCWs that they know quite often what to do e.g. open windows (to allow for ventilation), but were not able to do so (windows don't open).

Training is crucial to ensure that HCWs are capable of practicing TBIPC. Training should include auxiliary staff because they often assist in patient related activities such as prioritizing patients with cough. Furthermore, other HCWs and facility management should recognize and appreciate this role.

Our study also showed that existing practices in healthcare facilities form barriers to adequate use of TBIPC measures such as prioritization of patients with cough. Usually patients arrive early at healthcare facilities and staffs attend to them on a ‘first-come-first-serve’ basis. The fact that some patients are pretending symptoms to get access to prioritized attendance probably demonstrates that there are long queues in healthcare facilities. The long waiting times are an additional burden for patients who have to get to work or attend to other responsibilities. It could be that using an appointment system would reduce waiting time for patients and make prioritization measures more acceptable.

The use of masks by patients [12] and the use of respirators by HCWs have an alienating or depersonalizing effect and reduce the HCWs' ability to provide compassionate care. Wearing a mask by patients or a respirator by HCWs should become acceptable to both. The Ugandan TBIPC guidelines make a clear effort to change existing practice: “They need to understand that HCWs may wear personal protective equipment (respirators) sometimes, or that patients may be asked to wear a mask in order to protect others. Safety without stigma should be the goal. A request to wear a mask or provide sputum outside the healthcare facility or in a well-ventilated room should not be stigmatizing, but is part of a safer clinic for everyone.” [13]

Participants also found respirators uncomfortable to wear, especially in high temperatures. This is very relevant for Mozambique and similar climate settings where temperatures are often high. For example, in Tete province temperatures during summer may raise to 45°Celsius and above. These high temperatures may contribute to inadequate use of the respirators.

Participants were creative in using coping strategies for the difficulties they faced in using TBIPC measures, however, these strategies may diminish the efficacy of measures. Not all HCWs may be able to provide their own protection material. Opening the respirator will reduce its efficacy. Healthcare management should take the responsibility to protect HCWs through provision of the necessary material.

Equally or even more importantly is motivating HCWs to implement TBIPC measures. Motivation among HCWs is already a challenge in the Mozambican healthcare system [14]. Reasons are low pay, inadequate work conditions, and lack of control over career development. Adding implementation of TBIPC measures, of which several are beyond the HCWs' control and which is potentially perceived as an additional workload, may negatively influence HCWs' motivation for TBIPC implementation. Therefore programs need to concentrate more on improving HCWs' motivation and offer support of colleagues and supervisors [15]. This also applies to auxiliary staffs, who were crucial in TBIPC implementation. Using behavior change communication and motivation techniques as part of the TBIPC trainings might be helpful.

Behavior is not always the consequence of perfect logic as principles of psychological theory suggest [16]. Social, emotional and environmental factors are all also relevant. For instance, improving workplace practice through ensuring that senior staff members follow guidelines and act as role models, may encourage junior workers to develop good practice from the start of their career [17]. Lastly, HCWs are probably more likely to follow guidelines if they feel ownership of these guidelines. Involvement of HCWs or representatives of their group in the development of the guidelines may create a sense of ownership [18].

### Limitations

Qualitative studies' findings may be extrapolated only with care, as they are often closely linked to local settings and circumstances. However, our study involved participants from several areas in Mozambique. Furthermore, our findings show similarity with studies from other Sub-Sahara African settings and apparently the identified issues surpass the settings in which they were identified. In addition, activities in TB programmes are quite standardized. We therefore think that Mozambique and similar settings may benefit from our findings to inform strengthening TBIPC.

Another limitation is that participants in FGDs may respond with socially desirable answers, especially if their seniors are present. Therefore we had the discussions by category of HCWs. However, in one group the local chief medical officer was present and the other participants may have given desirable responses. This did not affect the overall results of the study as the same issues came up in other groups.

## Conclusions

HCWs in Mozambique perceive a high occupational risk of TB infection and apply TBIPC measures to reduce this risk. They report several challenges in using these measures. Practical guidelines translated into workplace procedures, training, improving motivation of HCWs, support and supervision and creating new work practices may improve correct and consistent use of TBIPC. Besides, healthcare authorities should address healthcare system related improvements since these are beyond the control of the individual HCW.

Many resource-limited settings face a gap in the necessary funding for healthcare services including TB [1]. This is unlikely to resolve soon and future research should look into practical, low-cost and innovative solutions related to applying TBIPC in resource-limited settings. Because the acceptability of certain measures may vary across societies and communities [12], it is important to involve HCWs and healthcare facility users in this research.

## Supporting Information

S1 File Prompts used in the focus group discussions. This file contains the prompts used in the focus group discussions.
(DOC)
