# Supplementary material for: Healthcare Workers' Challenges in the Implementation of Tuberculosis Infection Prevention and Control Measures in Mozambique
Source: PLoS One. 2014 Dec 15;9(12):e114364. doi: 10.1371/journal.pone.0114364 (PMC4266607; doi:10.1371/journal.pone.0114364)
Supplement: S1 File — Prompts used in the focus group discussions. This file contains the prompts used in the focus group discussions. (DOC) [file pone.0114364.s001.doc]

## File S1. Prompts used in the focus group discussions. Supporting information of manuscript “Healthcare Workers’ Challenges in the Implementation of Tuberculosis Infection Prevention and Control Measures in Mozambique”

1. ***Prompt: We would like to know if you have knowledge of the existence of TB transmission in your health facility? If yes, could you please comment on this?*** Do you think TB transmission is a serious problem at your place of work? Could you explain why you think so?
2. ***Prompt: Is the risk of transmission during work as high as outside the work environment? Can you explain why you think so?*** Do you mind working in a situation where there is a risk of TB transmission?
3. ***Prompt: Do you know how to prevent TB transmission?*** What do you recommend to clients of the health facility in your day-to-day situation on how to prevent TB transmission? What is the most important group of clients for those recommendations? Do the clients follow the recommendations?
4. ***Prompt: Who in the health facility receives information about TB?*** Who do you think should receive information about TB??

1. ***Prompt: What measures do you take during work to prevent TB? Do you always take these measures? Can you explain why not or why you do so?*** Which measure(s) is(are) most important? What would be the three most important measures?
2. ***Prompt: What problems or difficulties do you run into, leading to not use TB prevention measures?*** Why is that so?
3. ***Prompt: Do other health care workers use the TB prevention measures? Please comment on your answer.***
